# 1416. The Economic Burden of Adverse Events Requiring Acute Care Services from Outpatient Parenteral Antibiotic Therapy (OPAT) Treatment

**DOI:** 10.1093/ofid/ofac492.1245

**Published:** 2022-12-15

**Authors:** Mauricio Rodriguez, Georgia Buscaglia, Steven Tolle, Darren Michael

**Affiliations:** Spero Therapeutics (Former Employee), San Antonio, TX; Forte Analytics, Denver, Colorado; Forte Analytics, Denver, Colorado; Forte Analytics, Denver, Colorado

## Abstract

**Background:**

Antimicrobial resistance (AMR) is a growing threat. ESBL-producing Enterobacterales infections are rising, especially within the community setting. Patients requiring OPAT services will increase based on AMR to oral antibiotic (ABX) options. OPAT adverse events (AEs) are linked to the use of IV catheters, followed by adverse drug events. Complications that arise from OPAT, often necessitate acute care services. We sought to quantify costs associated with OPAT AEs.

**Methods:**

A multicenter retrospective claims analysis from the state of Utah’s (UT) Public IBIS database was performed for 2020. OPAT AEs as described in the literature were used to query charges. All UT hospitals and common OPAT AE principal diagnosis (PDx) codes were included in the analysis. Estimated inpatient (IP) costs associated with common OPAT AEs were calculated from a charge-to-cost ratio (22.5%) using publicly available data from the Centers for Medicare & Medicaid Services. Event counts reported for UT were scaled proportionally to estimate total events for the U.S. population. Emergency department (ED) incidence rates for OPAT AEs from 2016 to 2020 were also examined.

**Results:**

During the study period, 248,843 patients met study inclusion for an OPAT AE PDx. Among IV-related complications, catheter phlebitis accounted for highest median cost per IP event at $14,051. Other PDx, included catheter blockage and central line-associated bloodstream infections at $11,237 and $10,103, respectively, followed by $9,371 for complications post injection. Thrombotic events equated to total of $11,915 for the combined costs of deep venous thrombosis and pulmonary embolism. Lastly, *C. difficile* infections accounted for a median cost of $5,284 (**Figure 1**). Age-adjusted rates of ED activity related to AEs rose to 17.6 per 10,000 in 2020; this marked an 18% increase from 2016 (**Figure 2**).

**Figure 1.**
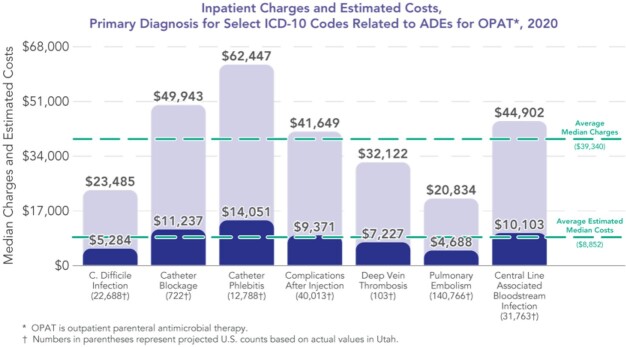

**Figure 2.**
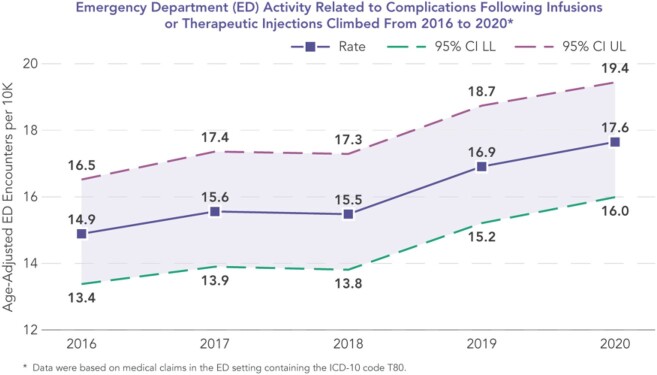

**Conclusion:**

Give that viable oral ABX treatment options in the community setting are limited, patients will require additional OPAT services as AMR rates continue to escalate. OPAT services are not without added risks of complications, as the average median cost for an OPAT AE was $8,852. These costs may be minimized by the addition of new oral ABXs that overcome AMR, thus improving patient outcomes.

**Disclosures:**

**Mauricio Rodriguez, PharmD, MS-HEOR, BCPS, BCCCP, BCIDP**, Spero Therapeutics: Employee **Georgia Buscaglia, PhD**, Spero Therapeutics: Advisor/Consultant **Steven Tolle, BFA**, Spero Therapeutics: Advisor/Consultant **Darren Michael, PhD, CC, SC**, Spero Therapeutics: Advisor/Consultant|Spero Therapeutics: Grant/Research Support.

